# Streamline pair selection for comparative flow field visualization

**DOI:** 10.1186/s42492-020-00056-8

**Published:** 2020-08-27

**Authors:** Shoko Sawada, Takayuki Itoh, Takashi Misaka, Shigeru Obayashi, Tobias Czauderna, Kingsley Stephens

**Affiliations:** 1 Ochanomizu University, 2-1-1 Otsuka, Bunkyo-ku, Tokyo, 1128610 Japan; 2 Tohoku University, 2-1-1 Katahira, Aoba-ku, Sendai, Miyagi 9808577 Japan; 3 Monash University, Wellington Road, Clayton, Victoria Australia

**Keywords:** Computational fluid dynamics, Streamline selection, Comparative flow field visualization, Virtual reality

## Abstract

Fluid dynamics simulation is often repeated under varying conditions. This leads to a generation of large amounts of results, which are difficult to compare. To compare results under different conditions, it is effective to overlap the streamlines generated from each condition in a single three-dimensional space. Streamline is a curved line, which represents a wind flow. This paper presents a technique to automatically select and visualize important streamlines that are suitable for the comparison of the simulation results. Additionally, we present an implementation to observe the flow fields in virtual reality spaces.

## Introduction

Computational fluid dynamics (CFD) has been recently applied in various academic and industrial fields owing to the evolution of high-performance computing and numeric simulation technologies. Simulation and visualization have played essential roles in numerically analyzing and simulating natural phenomena and in visually assisting the understanding of users. Computer visualization methods involve computer graphics and human-computer interaction techniques. They have been widely applied to represent the results of fluid dynamics computations.

Streamline is one of the most popular representations for visualizing flow fields such as volume datasets of CFD simulations. It forms a curved line via a set of segments, whose tangents are parallel to the flow vectors. We can effectively visualize the flow fields by generating an appropriate number of streamlines in a volume dataset. Furthermore, users can intensively represent the critical regions of the volume datasets by generating streamlines only in the critical regions. Automatic generation of appropriate number of streamlines is an essential problem. Therefore, many researchers have conducted studies to address this problem.

Comparative visualization is another important problem for visualization of flow fields. Simulation experts in fluid dynamics often repeat their simulations as they adjust conditions. Furthermore, they often compare many simulation results. Comparative visualization assists in understanding the fluid phenomena and improving the accuracy of fluid simulations because users can effectively compare the results. Many comparative visualizations for scalar fields have been already presented. However, comparative visualization for flow fields still remains an open issue.

We previously presented a comparative visualization technique [1], which overlays streamlines generated from each of the volume datasets as CFD simulation results. This technique generates streamlines in each of the volume datasets from the same seed positions. However, the seed positions were manually specified in this study because we did not implement an automatic mechanism of the streamline generation process.

This paper presents a comparative visualization technique for flow fields featuring an automatic streamline selection method. The technique generates a large number of streamlines in an entire volumetric region of a CFD simulation, and then selects a user-specified number of meaningful streamlines. This representation assists users to effectively discover differences in the flow fields due to the changes in the conditions.

This paper also presents a virtual reality (VR) application, which displays the streamlines selected by our method, to provide an environment for immersive observation of important streamlines. We expect that VR environments can solve the problem of sufficiently recognizing fluid phenomena by using a two-dimensional (2D) display to observe three-dimensional (3D) fluid simulation results.

Several studies on streamline selection have been conducted to evaluate geometry and screen space density of streamlines. However, these ideas have not been applied to comparative visualization. Meanwhile, there have been several studies on ensemble vector field visualization that evaluate distances or differences among streamlines generated from different datasets. The main contribution of our study presented in this paper involves the development of streamline-based comparative visualization by considering all the aforementioned factors: geometry and screen space density of streamlines, and distances or differences among streamlines generated from different datasets. This paper introduces visualization examples and numerical results that depict different sets of streamlines due to a combination of the aforementioned factors. Furthermore, the paper introduces user evaluation results.

The remainder of this paper is organized as follows. The rest of this section lists related extant studies and discusses the novelty of the presented technique with respect to the extant studies. We present the technical details of our proposed technique for selecting meaningful streamlines in Section methods for automatic streamline selection. Section results and discussion introduces the visualization examples. Finally, we present our conclusions and discuss future research in Section conclusion and future work.

In the rest of this section, we introduce existing studies on streamline selection and comparative vector field selection. Furthermore, we explain the basis of our study and our previous study for applying VR technologies to flow visualization.

### Streamline selection

Many techniques have been developed for automatic streamline selection in a single CFD simulation result. The study by Mattausch et al. [2] is one of the first studies on streamline selection in 3D spaces.

Density-controlled seed point setting has been an important problem for streamline-based vector field visualization. McKenzie et al. [3] discussed space partition schemes to optimize seed point setting and demonstrated the importance of this idea in streamline generation. Liu et al. [4] applied the centroidal Voronoi tessellation method for the specification of seed points in streamlines.

Geometry-based streamline selection is another interesting approach. Chen et al. [5] presented a streamline selection technique by considering the geometric similarities among the streamlines. Wei et al. [6] presented a sketch interface to interactively specify streamlines along the hand-drawn shapes.

Furthermore, view-dependent factors for streamline selection have been discussed to a significant extent. Lee et al. [7] proposed a visualization method that recommends the best viewpoint where streamlines projected onto a screen are highly evaluated. They suggested that poor choices of viewpoints may damage the comprehensibility of flow fields when too many streamlines are displayed. Ma et al. [8] proposed another method to select streamlines by calculating the view-independent and view-dependent importance of streamlines with the application of an interactive viewpoint manipulation mechanism. Tao et al. [9] simultaneously solved the problem of streamline and viewpoint selections by applying the concept of the information channel.

Furuya and Itoh [10] presented a technique for simultaneous visualization of scalar and vector fields. They presented a streamline selection technique that preserves high comprehensibility of flow fields by determining occlusion by isosurface. The technique presented in this paper is inspired by this streamline selection technique.

The aforementioned studies mainly focus on selecting streamlines that exhibit informative geometry and simultaneously considering view-dependent appearance factors. However, these studies have not been applied to comparative visualization. We applied these ideas for comparative vector field visualization.

### Comparative vector field visualization

The study of comparative scientific visualization has a long history [11]. Specifically, many studies on comparative scalar field visualization have been conducted. For example, Lampe et al. [12] presented a visualization technique, which enables comparison among volume datasets that exhibit different structures.

Furthermore, comparison of vector fields in the volume datasets has been an active topic of research, especially for the representation of ensemble vector fields. Hummel et al. [13] applied the evaluation of individual and joint transport variance to flow maps of the ensemble vector fields.

Whitaker et al. [14] innovated a new visual representation, termed as contour boxplots, for ensemble visualization.

Guo et al. [15] and Liu et al. [16] presented line-based representation of ensemble vector fields. The ideas in these studies are similar to our ideas presented in this paper because they evaluated differences between flow lines. Specifically, Guo et al. applied Euclidian distances, while Liu et al. applied the longest common subsequence distance. However, these studies did not discuss view-dependent appearance factors.

### Comparative visualization with streamlines

The starting point of our study presented has already been introduced by Hattanda et al. [1]. They presented a software prototype to comparatively and interactively visualize flow simulation results. This study visualizes a pair of volume datasets of the flow fields as the results of two CFD simulations under different conditions. The prototype provides a user interface to interactively specify seed points in the 3D space and generates schematic of streamlines in two colors that are generated in each of the volume datasets. In this study, we visualized a pair of simulation results at Haneda International Airport. In the simulation, the wind flow is varied, which in turn changes the condition of the simulations. However, the seed points of streamlines were individually manually set by the users of the software. Therefore, the users were required to possess knowledge and experience for setting appropriate sets of seed points, and this was problematic.

### Visualization with VR technologies

VR technologies have been applied for visualization in the field of science and technology for a long time [17]. Specifically, visualization of 3D vector fields [18] is a good application of the VR technology. For example, Forsberg et al. [19] presented a VR system that simulates transplantation of the blood flow. Coffey et al. [20] proposed a visualization tool that realized compatibility between overview and detail by using 3D and multi-touch display technologies. Thus, the aforementioned methods aid in observing the entire 3D visualization datasets in the VR space.

## Methods for automatic streamline selection

In this section, we describe the technical details of the proposed automatic streamline selection method for comparing a pair of CFD simulation results. Subsequently, in this section, we introduce our VR application that provides an environment for immersively observing the differences in the streamlines. The method generates two streamlines, which are termed as “streamline pair” in this paper. The streamlines are generated at the same seed position in each of the volume dataset pairs as simulation results with different conditions. This implies that the automatic streamline selection proposed in this paper is the automatic selection of streamline pair sets. Here, we assume that seed positions of streamlines are selected from all grid-points in the 3D space.

### Processing flow for automatic selection

**Fig. 1:**
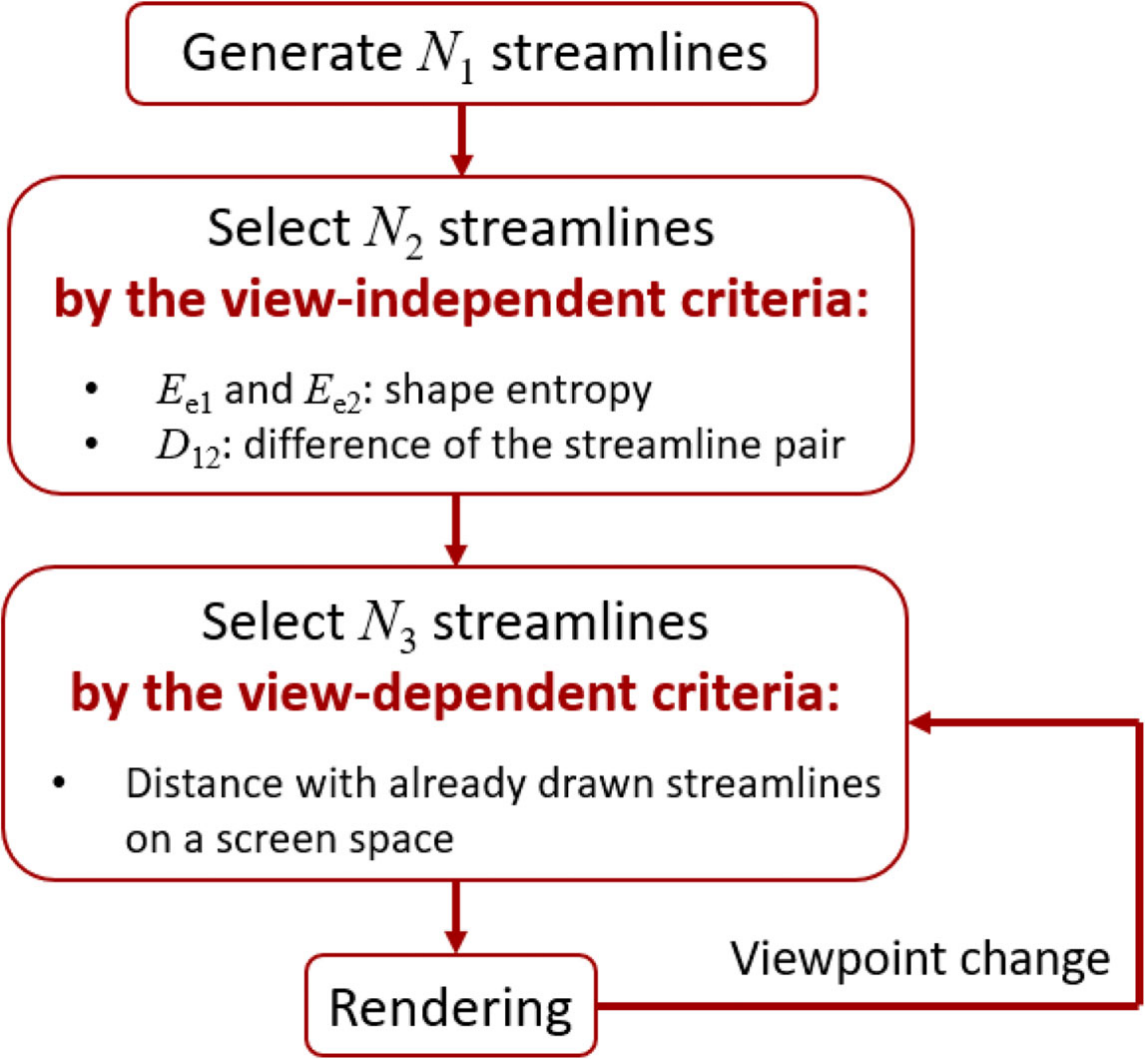
Processing flow of the presented technique

The processing flow of our proposed method is shown in Fig. 1. First, this method generates N_1_ streamline pairs in the entire 3D space for trial purposes. Our implementation randomly selects N_1_ grid-points as seed points to generate the streamline pairs. Next, the method calculates the following two values for each of the streamline pairs:

- shape entropy E_e1_ and E_e2_
- difference between the streamline pair D_12_

The method calculates a view-independent score S for each of the streamline pairs. Then, the upper N_2_ (N_1_ > N_2_) streamline pairs are extracted as meaningful streamline pairs based on their S values. Subsequently, N_3_ (N_2_ > N_3_) streamline pairs are selected when a viewpoint is fixed. The method reselects N_3_ streamline pairs whenever the viewing orientation is updated. Furthermore, our implementation approximates streamlines as polygonal lines consisting of short line segments generated inside elements of the volume datasets.

### View-independent evaluation for streamline pair selection

We define the score S as the linear combination of shape entropy and difference between a streamline pair in the equation below, where α satisfies 0 ≦ α ≦ 1:

$$ \mathrm{S}=\upalpha \left({\mathrm{E}}_{\mathrm{e}1}+{\mathrm{E}}_{\mathrm{e}2}\right)+\left(1\hbox{-} \upalpha \right){\mathrm{D}}_{12} $$

Our implementation calculates S as pre-processing only once when a volume dataset is provided.

#### Shape entropy

We applied the shape entropy defined by Ma et al. [8]. This was originally proposed to select streamlines from a single volume dataset. However, long and undulated streamlines are preferentially selected by applying this definition. We assume that this definition is reasonable because short streamlines are usually less informative, and straight streamlines do not usually represent interesting phenomena.

Specifically, the following equation is used to calculate the information entropy:

$$ {E}_{ei}=-\sum \limits_x\left(p(x)\log (x)\right) $$

Where, i is 1 or 2, and *p*(*x*) denotes the probability function of the properties of the vector field values of the sample points along the streamline.

Our implementation constructs a 2D histogram that represents the distribution of directions and magnitudes of the vector field values. The function *p*(*x*) is computed as the normalized bin count of the 2D histogram. Ma et al. [8] describes the detail of this function *p*(*x*) in their study.

This method calculates E_e1_ and E_e2_ independently.

#### Difference between a pair of streamlines

We preferentially extract a pair of streamlines that exhibit relatively different shapes.

Based on this preference, the proposed method calculates the difference between a pair of streamlines, D_12_, by calculating distances between vertices of the streamline pairs. The method matches vertices of a pair of streamlines one-by-one. Our implementation specifies the vertex closest to the vertex of the other streamline to coupled vertices.

Subsequently, the proposed method calculates D_12_ as the average of the distances between each of the coupled vertices via the following equation:

$$ {D}_{12}=\frac{1}{n_p}\sum \limits_{i=0}^{n_p-1} dist\left({\boldsymbol{p}}_{\boldsymbol{i}\mathbf{1}},{\boldsymbol{p}}_{\boldsymbol{i}\mathbf{2}}\right) $$

Where, *n*_*p*_ denotes the number of matched pairs of vertices, dist(**a**, **b**) denotes the distance between two positions **a** and **b**, and **p**_ij_ denotes the position of the i-th vertex of the j-th streamline.

**Fig. 2:**
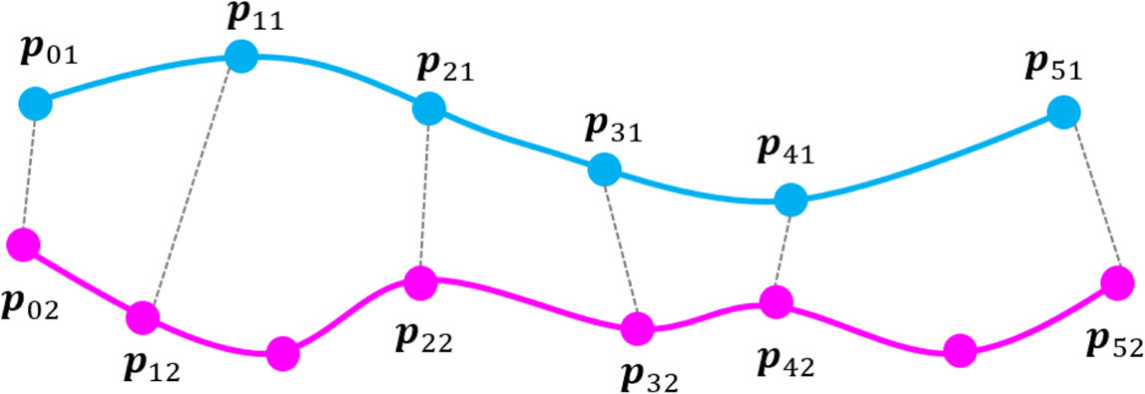
Coupling vertices to calculate the difference between a pair of streamlines. Pink curve line and cyan one are a pair of streamlines. Black dot is a seed point of this pair of streamlines, and gray dots are vertices of streamlines

Figure 2 illustrates the process of coupling vertices of a pair of streamlines.

### View-dependent streamline pair selection

In this study, we aim to avoid selection of a set of streamline pairs, which lead to many overlaps on the screen space. Several studies on view-dependent streamline evaluation of a single CFD simulation result have been presented. Furthermore, several studies on streamline selection that apply shape entropy have been presented. We apply the view-dependent streamline selection method proposed by Furuya and Itoh [10] to selectively display streamline pairs.

The technique displays streamline pairs in the order of the scores. Thus, meaningful streamlines are preferentially displayed. In this implementation, the process skips streamline pairs, which are significantly overlapped with already displayed streamlines. After the most important streamline pair is displayed, we repeat the following steps in a descending order from the second-place streamline pair.

1. Extract vertices of the current streamline. Let vertices V = {v_1_, v_2,_ ..., v_Nv_}.
2. Calculate the minimum distance to vertices of the already displayed streamlines from each of the extracted vertices v_i_.
3. Unselect the current streamline if it has more than a constant number of vertices of already displayed streamlines whose distances are less than the pre-defined threshold d_min_.

This process is repeated until N_3_ streamline pairs are drawn.

### Visualization in a VR space

We developed a VR environment to assist the immersive observation of the selected sets of streamlines. Although our streamline selection method considers view-independent and view-dependent conditions, the comprehensibility of the visualization results may not be satisfactory in 2D display devices when many streamline pairs are selected in a particular part of a 3D space. Thus, this may lead to a lack of understanding of flow phenomena. Therefore, our main motivation to develop a VR environment involves displaying the streamline selection results to ensure that a more appropriate and detailed comparison of flow dynamics simulation results can be realized.

**Fig. 3:**
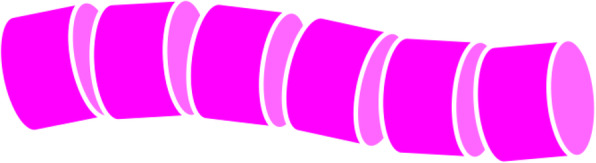
Example of a streamline drawn as connected cylinders

Our implementation of the automatic streamline selection (developed with Java) outputs the sets of selected streamlines into a specific format of text files. This implementation has a graphical user interface to display the selected streamlines. Furthermore, we developed a VR environment with Unity and Oculus Rift. Snapshots that display a set of selected streamlines in a VR environment are presented introduced in the next section. The Unity application illustrates streamlines as connected cylinders, as shown in Fig. 3.

The implementation on the top of the Unity application features an animated display showing the movement of the viewpoint along a user-selected streamline. This animation aids users in gaining a better understanding by comparing streamlines during the experiences of the undulation of the flow. We expect the users to look at the surroundings from the start-point to the end-point of the streamline. This Unity application supports a variety of manipulations, including viewing operations, such as rotation, scaling, and shifting, as well as the interactive streamline selection. We implemented these manipulations with an Xbox controller and also with a mouse and keyboard.

## Results and discussion

### Flow simulation applied to our case study

**Fig. 4:**
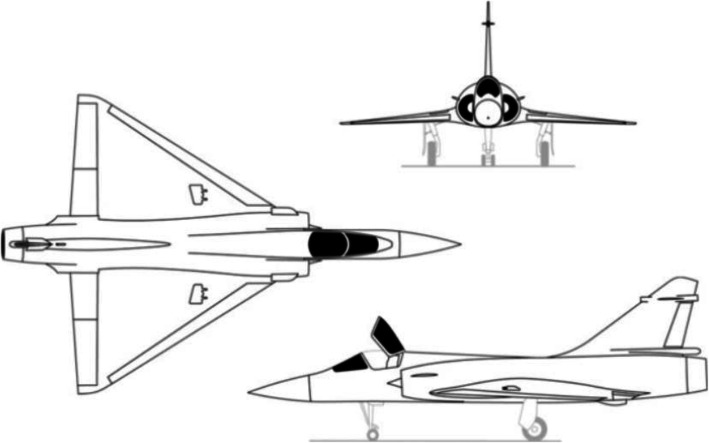
Example of an airplane that haves Delta wings (Mirage 2000C). See http://www.wikiwand.com/

We applied the proposed streamline selection technique to the fluid dynamics simulation results [21] for the geometric model of a “Delta wing”. The Delta wing is a delta-shaped wing of an airplane, as shown in Fig. 4.

**Fig. 5:**
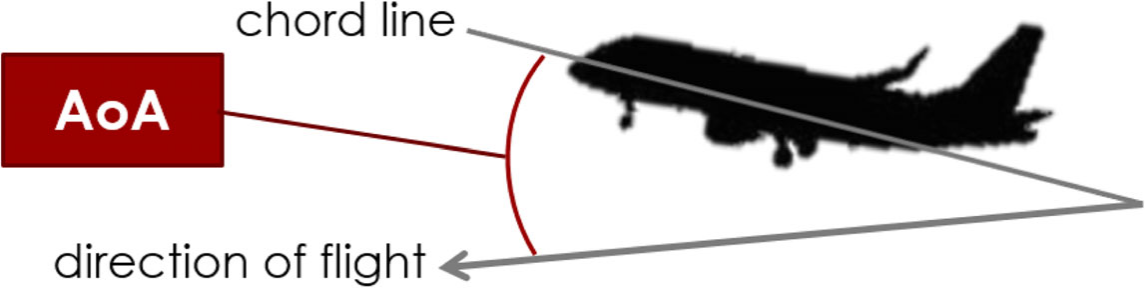
The red angle shows the angle of attack

We adjusted the angle of attack to conduct the simulations under different conditions. Figure 5 illustrates the angle of attack, which is an angle between the chord line of the wing and direction of the flight. This implies that the angle of attack is the inclination of the airplane with respect to the direction of the flight.

We conducted flow dynamics simulations by using the Tohoku University Aerodynamic Simulation and Building Cube Method codes. We set the angle of attack values as 20, 27, 30, and 33. We converted the simulation results to regular grid volumes that have 200 × 100 × 200 grid-points.

**Fig. 6:**
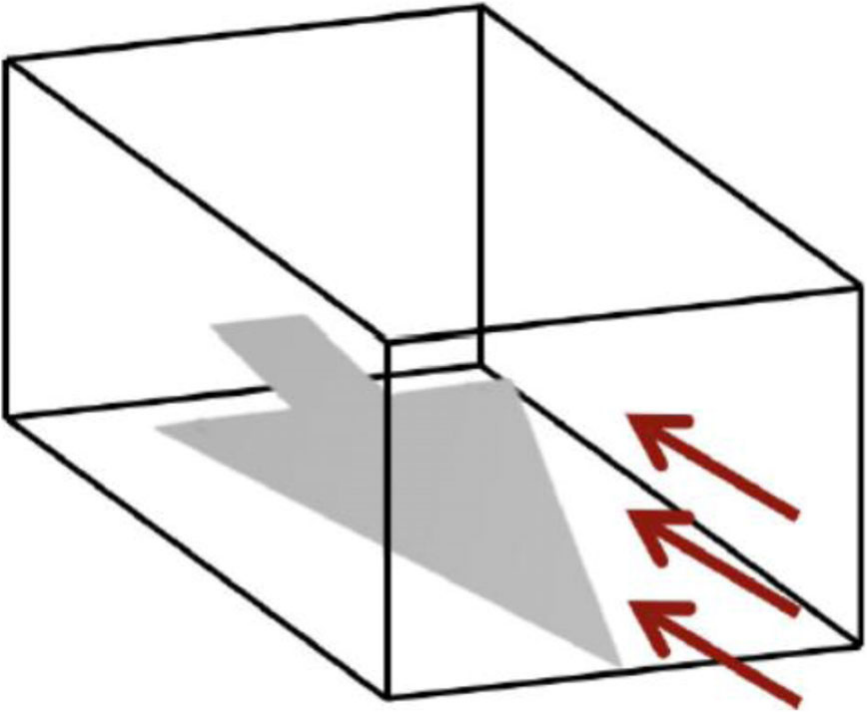
3D space of flow simulation

Figure 6 illustrates the 3D space for flow simulation. We introduced a wing model at the bottom of the 3D space, as illustrated by the gray triangle shown in the figure. Subsequently, we set a constant wind, as illustrated by the red arrows. We did not rotate the wing. We adjusted the direction of the wind to change the angle of attack.

The Delta wing exhibits good properties at supersonic speeds. Conversely, its aerodynamic characteristics during takeoff and landing are demanding. A higher angle of attack leads to more lift. However, a higher angle of attack may lead to dangerous vibrations of wings due to the backflow. Thus, a careful observation of the tradeoff between the flow simulation results with lower or higher angles of attack is required to determine an optimal design of the Delta wing.

### Visualization examples

**Fig. 7:**
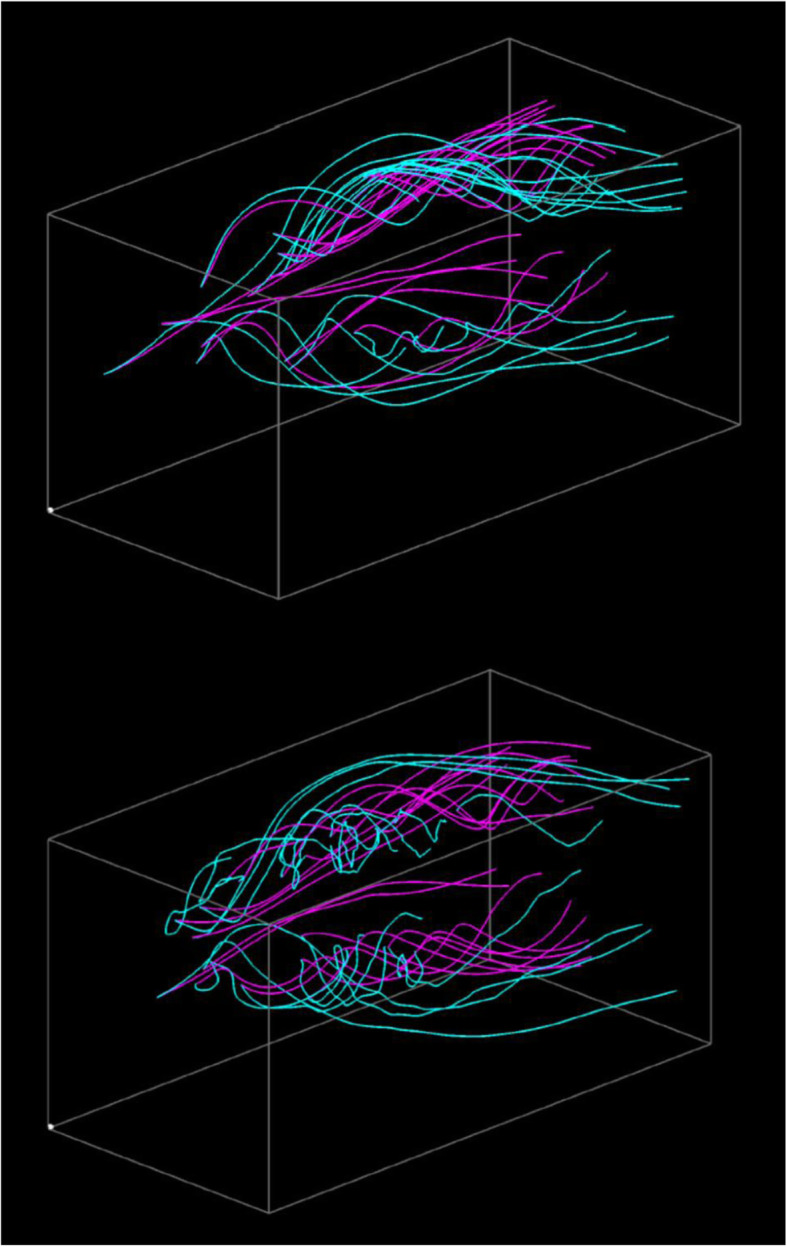
Visualization results. (Upper) The angles of attack are 20 and 27 degrees. (Lower) The angle of attack are 20 and 33 degrees

Figure 7 shows visualization results for applying the aforementioned volume datasets. The wing model was located at the left-back of the 3D space, and the wind was flowing from the left-front to right-back. The streamlines drawn in pink were generated from the simulation result with an angle of attack of 20 degrees. Furthermore, the streamlines drawn in cyan were generated from the results with an angle of attack of 27 and 33 degrees. Both visualization results show that streamlines are intensively generated around the wings, and the streamlines represent the vortices separated from the leading edge.

**Fig. 8:**
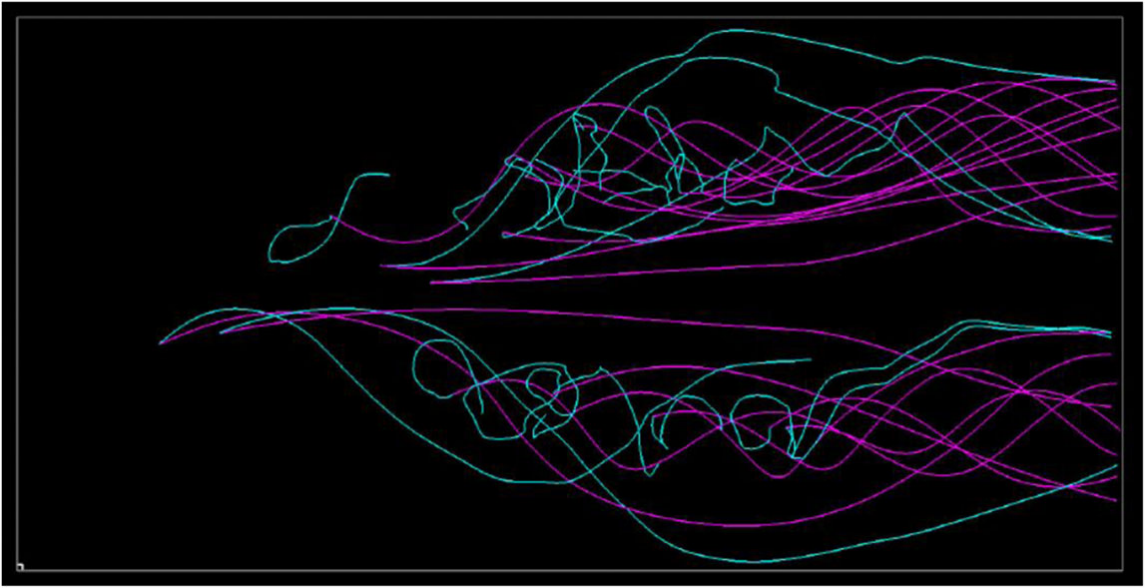
Visualization from another viewpoint. (20 and 33 degrees of the angles of attack)

Figure 7 (Upper) shows that several straight streamlines drawn in pink are selected, while many streamlines drawn in cyan are moderately swelled. The visualization represents the small differences between the simulation results at an angle of attack of 20 and 27 degrees. Furthermore, Fig. 7 (Lower) shows that several streamlines drawn in cyan are strongly swelled. Figure 8 shows the streamline selection result in Fig. 7 (Lower) rendered from another viewpoint. The view direction is parallel to the major normal vector of the wing, and the wind is from the left end of this figure. We can find the backflow and vortex breakdown from the streamlines drawn in cyan. They suggest that an angle of attack of 33 degrees can lead to a dangerous situation.

**Fig. 9:**
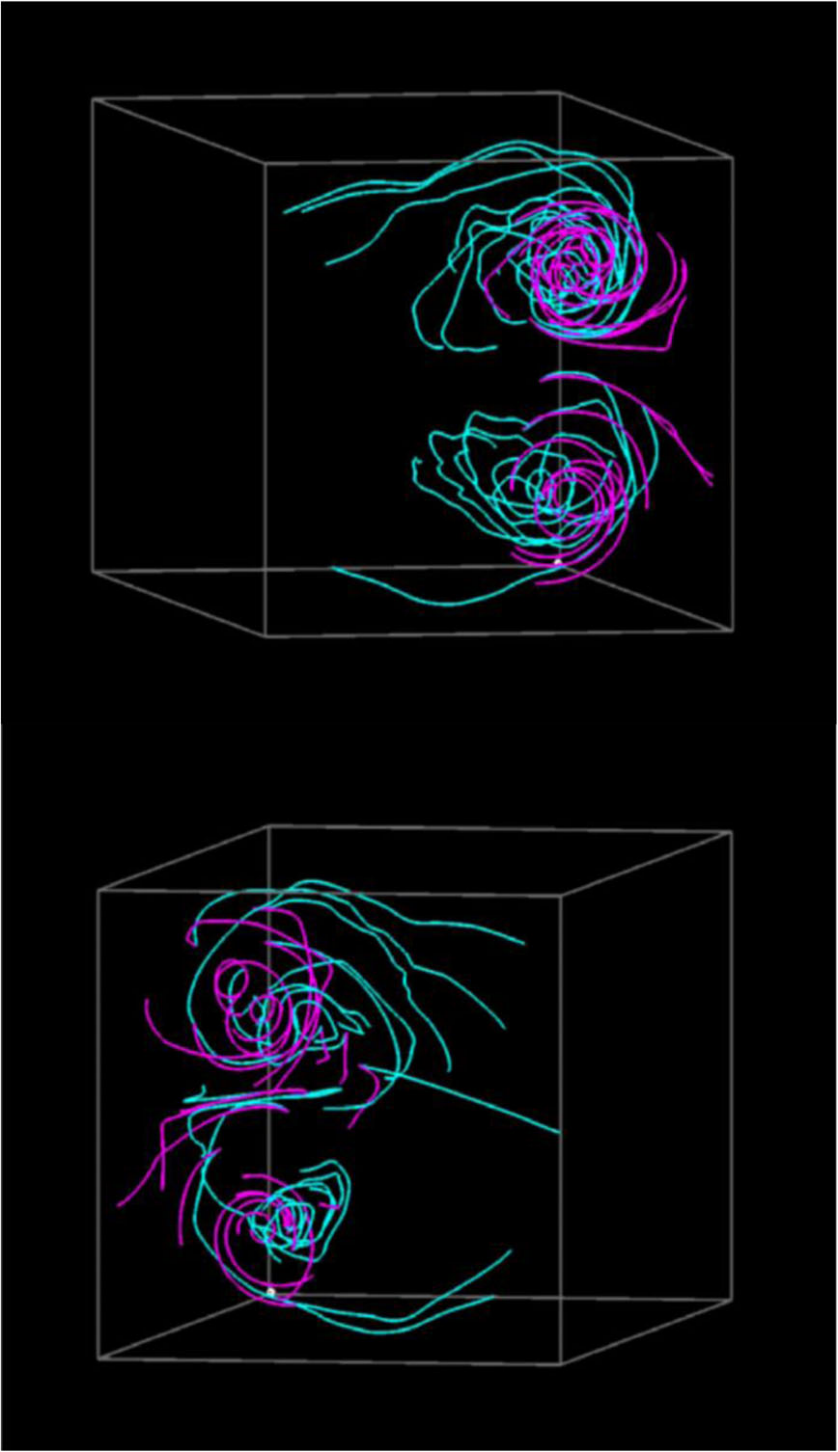
Streamline reselection after the update of the viewpoint

Figure 9 shows the effectiveness of the reselection of streamline pairs with the change in the viewpoint. Figure 9 (Upper) is an example that shows that the streamline pairs are first selected and then the viewpoint is updated. Figure 9 (Lower) shows the reselection of the streamline pairs after fixing the viewpoint. The reselection result leads to fewer overlaps among the streamlines due to the view-dependent streamline selection, which avoids overlap with already drawn streamlines.

Our technique calculates the score of streamlines as a weighted linear combination of the geometric entropy E_i1_ + E_i2_ and distance of streamline pair D_i_. We can adjust the weight α to achieve the preferable selection of streamline pairs. This is the main feature of the proposed technique when compared with the existing techniques.

**Fig. 10:**
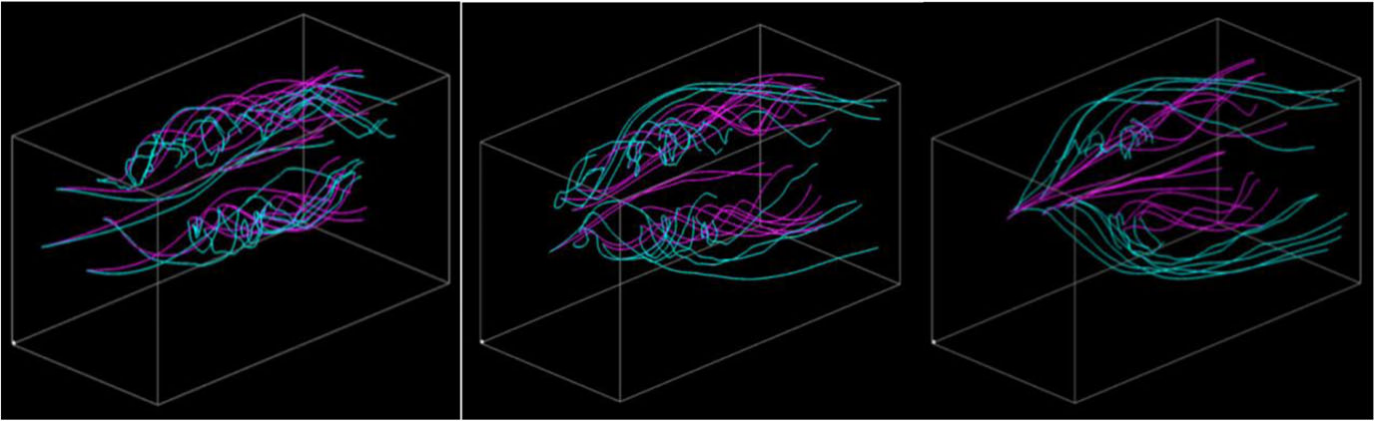
Visualization with various α values. (Left) Only the geometric entropy is applied (α = 1.0). (Center) Both the geometric entropy and distance of streamline pair is applied (α = 0.5). (Right) Only the distance of streamline pair is applied (α = 0.0)

Figure 10 shows visualization examples with various α values (α = 1.0, 0.5 and 0.0). This result demonstrates that different α values lead to different sets of streamlines while weighting the geometric entropy or distances of streamline pairs.

We calculated the sum of geometric entropy E_sum_ and the sum of distance D_sum_ of the selected streamline pairs. We repeated the streamline generation and calculation of E_sum_ and D_sum_ ten times for each value of α, excluded the maximum and minimum values of E_sum_ and D_sum_ in the ten results, and calculated the average of the remaining eight values of E_sum_ and D_sum_.

Table 1 shows the average E_sum_ and D_sum_ values with respect to different α values. The result shows that our implementation can effectively balance the geometry of streamline pairs and differences in streamline pairs by adjusting the α value. Thus, the result shows that a higher α leads to a higher E_sum_. This implies that the geometry of streamlines is prioritized, while lower α leads to higher D_sum_, thereby implying that the differences in streamline pairs are well represented.

**Table 1:** Average values of geometric entropy and distances of the selected streamline pairs

Average	α = 1.0	α = 0.75	α = 0.5	α = 0.25	α = 0.0
E_sum_	7.8309	7.7930	6.9106	6.6976	6.4407
D_sum_	10.9284	20.7740	22.3414	22.6109	23.8245

### Examples with our VR application

**Fig. 11:**
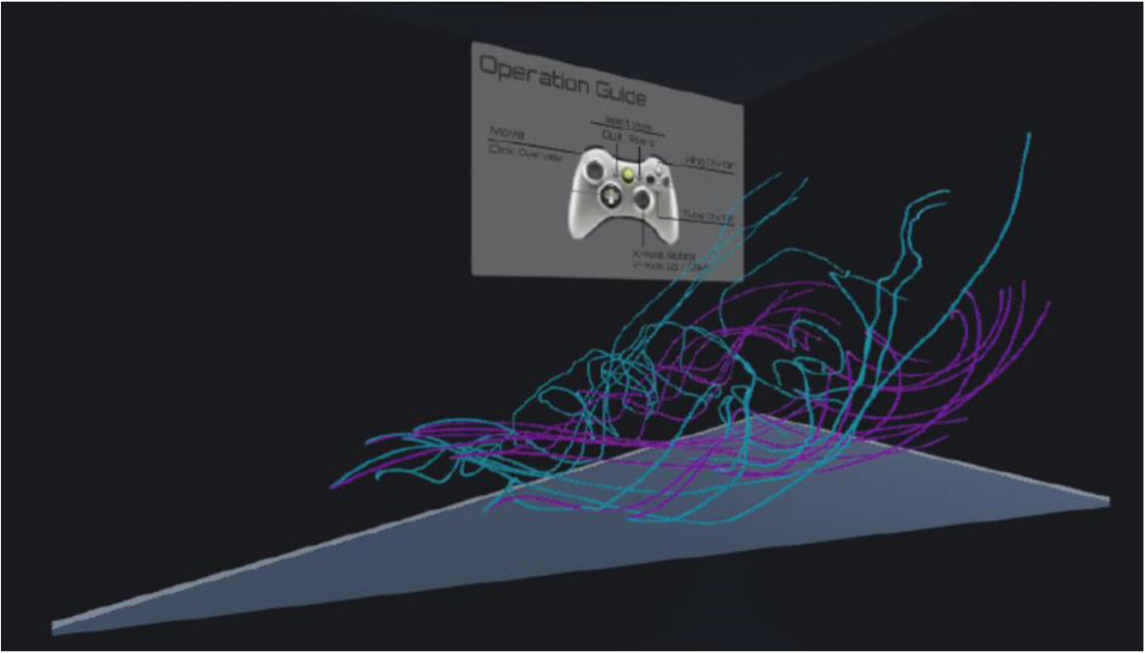
Example of streamlines in a VR space

**Fig. 12:**
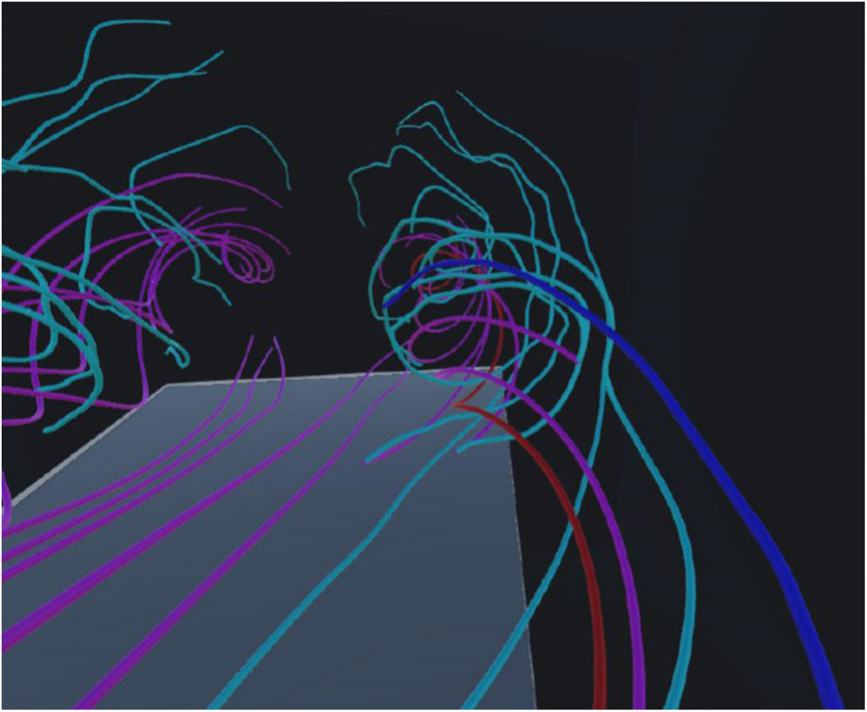
Animation along a user-selected streamline

Figure 11 shows an example of streamline selection results in a VR space. Streamlines denoted in pink are generated from the simulation result with an angle of attack of 20 degrees, while those indicated in cyan are from the result with an angle of attack of 33 degrees. Figure 12 shows another example of a snapshot of the animation when the viewpoint moves along a user-selected streamline. Specifically, the selected streamline and its pair are denoted in different colors as follows: pink to red and cyan to blue. We expect that the immersive environment assists users in observing differences in flow fields in detail and understanding fluid phenomena.

### User evaluation

We conducted user experiments to demonstrate the effectiveness of the presented technique. For these experiments, we invited 15 university students majoring in computer science.

#### Evaluation of streamline pair display in the same 3D space

We conducted a comparative user evaluation to demonstrate the effectiveness of the streamline pair display. We prepared two sets of flow visualization modules as follows: a set displays streamline pairs in the same 3D space as we presented in the study, and the other set displays streamlines generated from different volume datasets in the different display spaces. We applied two volume datasets with angle of attack values of 20 and 33 degrees in the experiment.

**Fig. 13:**
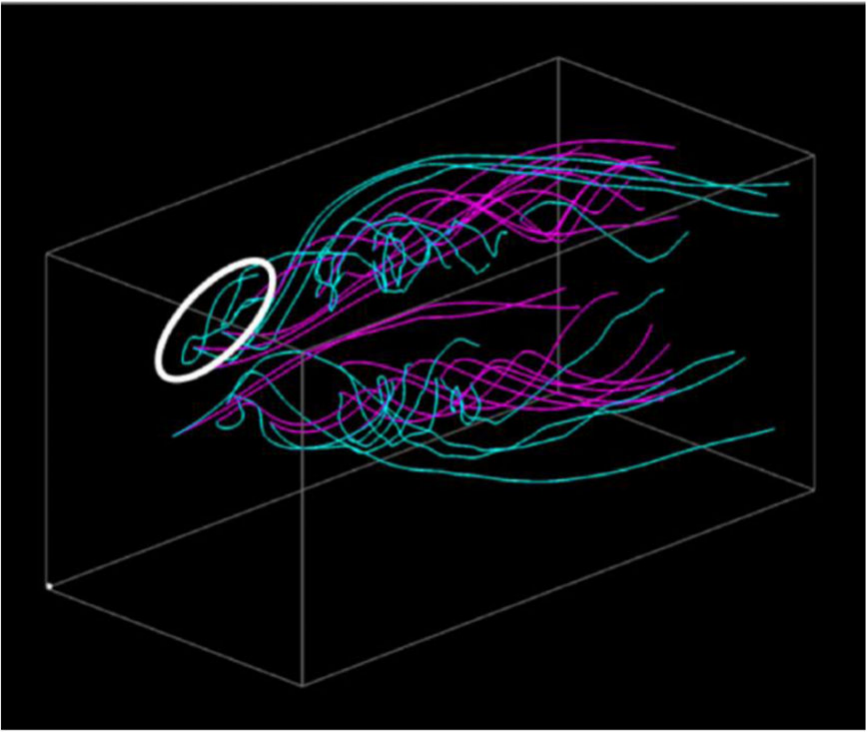
Visualization example that displays streamline pairs in the same 3D space

**Fig. 14:**
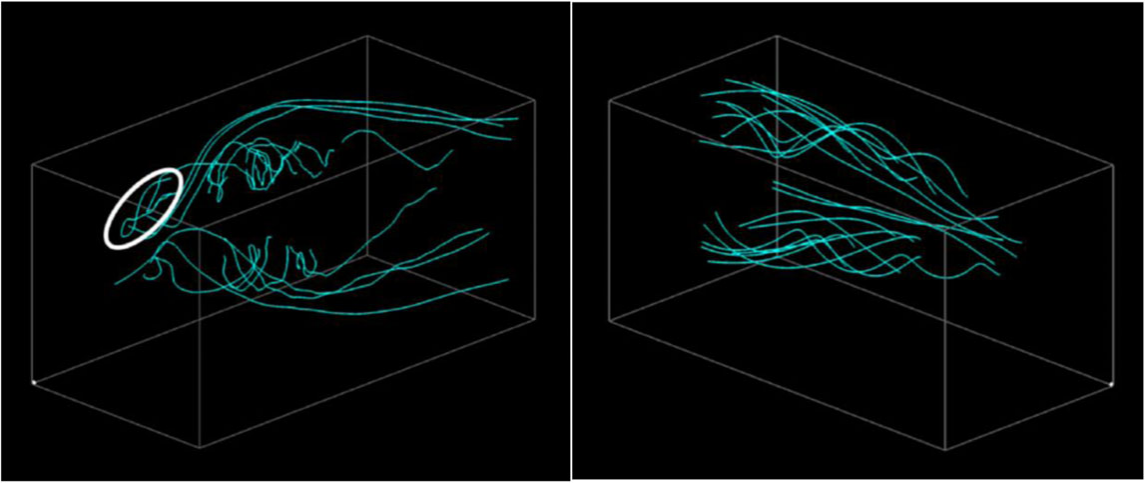
Visualization example that displays streamlines generated from the different datasets in the different display space

Figure 13 shows a visualization result that overlays the streamline pairs in the same 3D space, and this is termed as “overlaid visualization” in this section. Figure 14 shows another visualization example of the same datasets where streamlines generated from the different datasets are denoted in different display spaces and are termed as “arranged visualization” in this section. The white circles in Figs. 13 and 14 indicate that it is possible to determine the backflow in the volume dataset with an angle of attack of 33 degrees.

We asked participants to determine the types of streamlines that are selected from the volume dataset with an angle of attack of 20 degrees.

We generated two sets of streamline pairs that are termed as “Set A” and “Set B” in this section. We divided the participants into two groups with seven participants in “Group A” and eight participants in “Group B”. We asked members of each of the groups to perform the following tasks:

- [Group A]: First, observe the visualization result of Set A using overlaid visualization and answer the question. Then, observe the visualization result of Set B using arranged visualization and answer the question.
- [Group B]: First, observe the visualization result of Set A using arranged visualization and answer the question. Then, observe the visualization result of Set B using overlaid visualization and answer the question.

Table 2 shows the total number of participants, who provided appropriate answers. The result indicates that the effectiveness of the overlaid visualization proposed in the study exceeds that of the arranged visualization.

**Table 2:** Total number of participants who accurately explained the types of streamlines that are selected

Group	Overlaid	Arranged
Group A	22	20
Group B	24	18

We also asked all the participants to evaluate the overlaid and arranged visualizations based on the following aspects:

- the ease of understanding of fluid phenomena, and
- convenience of interactions while exploring the difference in flow fields

Based on a five-point Likert scale, we asked participants to associate the points as follows:

- 5: Strongly support overlaid visualization
- 4: Moderately support overlaid visualization
- 3: Even
- 2: Moderately support arranged visualization
- 1: Strongly support arranged visualization

Table 3 shows the statistics of the user evaluation.

**Table 3:** Total number of answers of participants based on the five-point Likert scale

Answer	1	2	3	4	5
Fluid phenomena	8	0	3	0	4
Difference of flow fields	8	3	0	4	0

The statistics suggest that half of the participants answered that arranged visualization is better or both are even in terms of the ease of understanding. This is because overlaid visualization draws twice the streamlines and therefore appears more complicated. Additionally, many of the participants suggested that overlaid visualization is better in terms of convenience for reselecting streamlines along with the change in the viewpoint. The result demonstrates that the proposed visualization is especially preferable and convenient for interactively reselecting streamlines and repeating the visualization.

#### Evaluation of streamline evaluation schemes

Subsequently, we asked participants to evaluate the streamline selection results with different α values. We used the streamline selection results with different α values shown in Fig. 10 in the experiment.

We asked participants to select one of the streamline selection results that satisfy the following conditions:

- [Choice 1]: Appropriate in terms of representing the difference in the flow field.
- [Choice 2]: Appropriate in terms of representing the fluid phenomena.

Table 4 shows the statistics of the choices of the participants. It is important to note that some of the participants selected multiple results, and thus the total numbers of participants in the tables exceeded the actual number of participants.

**Table 4:** Total number of answers of participants for choices 1 and 2

Answer	α = 1.0	α = 0.5	α = 0.0
Choice 1	5	12	4
Choice 2	3	11	7

Specifically, as shown in Fig. 10 (Left), strongly undulated streamlines are well selected. However, differently generated streamlines are not well selected when we set α = 1.0 to simply consider the geometric entropy. The main reason is potentially that many participants did not support the result shown in Table 4. Furthermore, as shown in Fig. 10 (Right), differently generated streamline pairs are well selected. However, strongly undulated streamlines are not well selected when we set α = 0.0 to simply consider the difference in the flow field. The main reason is potentially that the participants did not support the result shown in Table 4. The undulated streamlines and differently generated streamline pairs are well-balanced when we set α = 0.5, as shown in Fig. 10 (Center). The result indicates that it is possible to obtain visualization results that simultaneously represent flow phenomena and difference in the flow fields by combining the evaluations of the geometric entropy and distances between streamline pairs to calculate the scores of streamline pairs.

## Conclusion and future work

The study proposed a technique for the selection of appropriate sets of streamline pairs. The technique assists in visual comparison of the results of two CFD simulations that are conducted under varying conditions. We also developed a VR application that supports immersive observation of the streamline pairs.

There are future issues in terms of the extension of our implementation. The following include typical implementation issues.

Our current implementation simply calculates the linear combination of the entropy E_e1_/E_e2_ and difference D_12_ as the definition of view-independent score S. We do not have sufficient experiences to justify if the evaluation is well-balanced between the representation of the entire flow field and emphasis wherein streamline pairs are differently shaped. It is potentially interesting to observe the change in streamline selection results with respect to the changes in the calculation of the score S.

Another issue involves seed point selection. First, we applied a purely random function to randomly select seed points. Subsequently, we applied blue-noise sampling. However, it was not possible to obtain improved results. It is interesting to discuss on how to obtain better sets of seed points as a future issue.

The current implementation limits the input volume datasets to only orthogonal regular grids that divide a 3D space into equally sized rectangular elements. A future study can support unstructured volumes consisting of triangular, pyramid, prism, and rectangular elements.

Another idea involves extending the implementation to support linked views with information visualization methods such as scatterplots. Many studies on volume and scientific visualizations developed linked view systems with information visualization methods [22–24]. Based on the aforementioned studies, we would like to visualize the distribution of streamline pairs by information visualization such that we can interactively control the selection of streamline pairs.

Finally, we are interested in implementing interactive manipulation mechanisms for our VR application. Many scientific visualization studies on VR systems implemented interactive streamline generation mechanisms [25, 26]. A hybrid approach with interactive streamline selection and automatic reselection along with the interactive selection can improve the satisfaction of users.

## Data Availability

The dataset applied in this paper is not open but authors can provide on demand.

## Abbreviations

CFD: Computational fluid dynamics
VR: Virtual reality
